# Avoidant/Restrictive Food Intake Disorder-Related Symptoms and Food Neophobia in Children with Food Allergy: A Mixed-Methods Study of Frequency Estimates, Diet Quality, and Food-Related Psychological Experiences

**DOI:** 10.3390/nu18142399

**Published:** 2026-07-22

**Authors:** Rita Nocerino, Giuseppina Rosolia, Teresa Rea, Silvio Simeone, Caterina Mercuri, Alessandra Agizza, Serena Coppola, Roberto Berni Canani, Laura Carucci

**Affiliations:** 1 Department of Translational Medical Science, University of Naples “Federico II”, 80131 Naples, Italy; agizza.alessandra@gmail.com (A.A.); serena.coppola3@unina.it (S.C.); berni@unina.it (R.B.C.); laura.carucci@unina.it (L.C.); 2 NutriTechLab Academic SpinOff, University of Naples “Federico II”, 80131 Naples, Italy; 3 ImmunoNutritionLab at the Ceinge Advanced Biotechnologies Research Center, University of Naples “Federico II”, 80131 Naples, Italy; 4 Department of Biomedicine and Prevention, University of Rome “Tor Vergata”, 00133 Rome, Italy; 5 Bachelor’s Degree Programme in Paediatric Nursing, University of Naples “Federico II”, 80138 Naples, Italy; lisagiusy77@gmail.com; 6 Department of Public Health, University of Naples “Federico II”, 80131 Naples, Italy; teresa.rea@unina.it; 7 Department of Clinical and Experimental Medicine, University of Catanzaro Magna Graecia, 88100 Catanzaro, Italy; silvio.simeone@unicz.it (S.S.); c.mercuri@unicz.it (C.M.)

**Keywords:** food allergy, ARFID, food neophobia, selective eating, children, mixed-methods, caregiver reassurance, Mediterranean diet, eating behavior

## Abstract

**Background**: Children with food allergy (FA) may develop eating difficulties that extend beyond medically required allergen avoidance, including food neophobia, selective eating, and avoidant/restrictive food intake disorder (ARFID)-related symptoms. This mixed-methods study aimed to assess ARFID symptoms, food neophobia, diet quality, and food-related psychological experiences in children with FA. **Methods**: Seventy children with confirmed FA were enrolled in an observational cross-sectional mixed-methods study conducted at a tertiary pediatric allergy center. Quantitative assessment included the Eating Disorders in Youth-Questionnaire (EDY-Q), the Italian Child Food Neophobia Scale (ICFNS), and the Mediterranean Diet Quality Index for Children and Adolescents (KIDMED). Qualitative data were collected from a purposive subsample of 26 children through semi-structured child-friendly interviews and drawing-based elicitation activities and were analyzed using inductive thematic analysis. **Results**: A positive EDY-Q screening result for ARFID-related symptoms was observed in 3 children (4.3%). Selective Eating showed the highest median EDY-Q domain score (median 1.50, IQR 0.67–4.00), followed by Food Avoidance Emotional Disorder (median 1.00, IQR 0.00–2.00) and Functional Dysphagia (median 0.00, IQR 0.00–2.00). Moderate food neophobia was observed in 45 children (64.3%), while 24 children (34.3%) were classified within the high food-neophobia category. KIDMED score was negatively correlated with EDY-Q total score (Spearman’s ρ = −0.302; *p* = 0.011), Selective Eating (ρ = −0.356; *p* = 0.002), and Functional Dysphagia (ρ = −0.238; *p* = 0.048). In an exploratory hierarchical regression, Selective Eating remained inversely associated with KIDMED after adjustment for demographic and FA-related covariates (β = −0.386; *p* = 0.002); however, the overall model did not reach statistical significance (*p* = 0.081), and the additional clinical covariates did not significantly improve model fit. Accordingly, the fully adjusted findings should be interpreted as exploratory. Qualitative analysis identified five themes: anticipatory fear of food and tasting, emotional memory of allergic reactions and generalization of risk, selective avoidance and food rigidity, reassurance-seeking and decision-making dependence, and ambivalent parental dynamics. **Conclusions**: Within this tertiary-care sample, positive EDY-Q screening results were uncommon, whereas broader food-related difficulties were identified. Pediatric FA assessment may benefit from attention to eating behavior, diet quality, and avoidance extending beyond confirmed allergens.

## 1. Introduction

Food allergy (FA) is an increasingly recognized chronic condition affecting up to 10% of children worldwide and representing a major public health challenge because of its negative medical, nutritional, psychological, social, and economic impact [1,2]. Recent Italian epidemiological data confirmed a progressive increase in pediatric FA incidence and prevalence between 2009 and 2021, particularly among children aged 0–3 years [3].

Current FA management requires accurate diagnosis, individualized dietary recommendations, prevention of accidental exposure, and maintenance of tolerated foods whenever possible in order to avoid unnecessary dietary restrictions and preserve nutritional adequacy [2,4]. However, this balance can be particularly challenging during childhood, when eating behaviors are strongly influenced by developmental, emotional, social, and family factors [5,6].

Beyond its medical and nutritional consequences, FA may also shape the emotional and relational context of eating. Children with FA and their families often experience reduced quality of life, fear of accidental reactions, and social limitations [5,7,8], while caregivers may develop hypervigilance and food-related concerns that affect daily decision-making [6,8]. Because allergen avoidance is medically necessary, vigilance represents an adaptive component of FA management. However, fear and uncertainty may coexist with, or extend beyond, appropriate safety behaviors. Conceptually, perceived food-related threat, emotional memory of previous reactions, sensory generalization of perceived risk, and caregiver-mediated reassurance may be associated with avoidance of tolerated, unfamiliar, or uncertain foods. This framework provides a rationale for examining eating behaviors that extend beyond medically required allergen avoidance, without presuming temporal or causal relationships [9,10,11].

In some children, avoidance may progressively expand from confirmed allergens to tolerated, unfamiliar, or uncertain foods, leading to excessive dietary restriction and distress around eating [10,11,12]. Distinguishing appropriate allergen avoidance from maladaptive avoidant/restrictive eating is clinically important because these behaviors may have different implications for growth, nutritional status, psychological functioning, and disease management [9,11].

Avoidant/Restrictive Food Intake Disorder (ARFID) is characterized by persistent food restriction resulting in nutritional, growth-related, or psychosocial impairment in the absence of body-image concerns [13,14]. Restrictive eating may be driven by low interest in food, sensory sensitivity, or fear of aversive consequences such as choking, vomiting, gastrointestinal discomfort, or allergic reactions [13,15]. Emerging evidence suggests that children with FA may be particularly vulnerable to fear-based ARFID pathways because repeated allergic reactions, food-related anxiety, and medicalized avoidance may reinforce restrictive eating patterns [11,12]. Recent systematic reviews have emphasized the uncertainty and methodological heterogeneity of the available evidence. Ciciulla et al. reported considerable variability in estimates of eating disorders among individuals with FA because of differences in study populations, definitions, and assessment instruments [9]. Similarly, Sánchez-Cerezo et al. identified substantial heterogeneity in the reported epidemiology of ARFID among children and adolescents [15]. These findings support the need to distinguish questionnaire-based screening from formal diagnosis and to interpret frequency estimates within the characteristics of the population studied.

Food neophobia, defined as reluctance to eat unfamiliar foods, represents another important dimension of eating behavior [16]. Although commonly observed during early childhood, persistent food neophobia may reduce dietary variety and compromise diet quality [17,18]. Recent evidence suggests that FA may be associated with increased food neophobia, altered food-related experiences, and reduced dietary flexibility, potentially because unfamiliar foods are perceived not only as novel but also as unsafe [10,18,19,20].

Although ARFID and food neophobia may both involve food avoidance and reduced dietary variety, they are partially overlapping but distinct constructs. ARFID is a clinical feeding or eating disorder requiring restrictive intake associated with significant nutritional, growth-related, supplementation-related, or psychosocial consequences, and may be driven by sensory sensitivity, low interest in eating, or fear of aversive consequences [13,14,15]. Food neophobia, by contrast, is a dimensional reluctance to try unfamiliar foods and may occur as a developmentally expected behavior without clinically significant impairment [16,17,18]. A child may therefore show high food neophobia while maintaining an adequate intake of familiar foods and not meeting ARFID criteria. Conversely, ARFID-related restriction may involve familiar foods or low interest in eating without marked neophobia. Thus, food neophobia is neither necessary nor sufficient for ARFID.

The relationship between FA, ARFID symptoms, and food neophobia is likely complex and bidirectional. Previous allergic reactions, fear of anaphylaxis, uncertainty regarding hidden allergens, and parental hypervigilance may contribute to the development and maintenance of avoidant eating behaviors [6,21]. Conversely, excessive food avoidance may reduce dietary diversity, impair QoL, and complicate clinical management, including food reintroduction, oral food challenges, and oral immunotherapy [4,12].

Despite growing interest in eating-related difficulties among children with FA, important knowledge gaps remain. Previous studies have used heterogeneous definitions and instruments and have not always clearly distinguished medically necessary allergen avoidance, food neophobia, ARFID-related screening findings, and formally diagnosed ARFID [9,10,11]. Moreover, few studies have simultaneously assessed ARFID-related features, food neophobia, and diet quality in children with confirmed FA, and even fewer have integrated standardized quantitative measures with children’s own accounts of food-related fear and avoidance. Addressing these gaps may help clarify how these related but distinct eating-related phenomena are experienced within pediatric FA care.

Because the relative frequency and overlap of these eating-related phenomena in pediatric FA remain uncertain, their simultaneous assessment is important. Eating-related difficulties may be expressed as a positive EDY-Q screening result for ARFID-related symptoms, as dimensional avoidant/restrictive eating features, as food neophobia, or as food-related fear and avoidance that do not meet the EDY-Q screening criteria for ARFID-related symptoms.

The present study was therefore not based on the assumption that ARFID would necessarily be frequent, but aimed to examine how these different and potentially overlapping patterns were distributed within a pediatric FA sample.

Therefore, the present mixed-methods study aimed to estimate the frequency of positive EDY-Q screening results for ARFID-related symptoms and to describe the distribution of food-neophobia categories within a tertiary-care sample of children with FA, while exploring the psychological experiences associated with food avoidance. By integrating quantitative and qualitative approaches, the study sought to provide a more comprehensive understanding of eating-related difficulties in pediatric FA and to identify factors that may support earlier recognition and targeted multidisciplinary assessment.

## 2. Methods

### 2.1. Study Design and Ethics

This study was designed as an observational, cross-sectional investigation with a mixed-methods approach, integrating quantitative and qualitative data within a single study design [22]. The study was conducted between August 2025 and February 2026 at the Pediatric Allergy Program of the University Hospital Federico II of Naples, Italy, a tertiary referral center for pediatric FA.

A convergent mixed-methods design was adopted according to established mixed-methods research principles [22], whereby quantitative and qualitative data were collected during the same study phase and subsequently integrated during interpretation to provide a more comprehensive understanding of avoidant/restrictive eating behaviors and food-related experiences in children with FA. Quantitative data were collected using questionnaires assessing ARFID-related symptoms and avoidant/restrictive eating features, food neophobia, and adherence to the Mediterranean diet [23,24,25,26].

Qualitative data were collected through child-friendly semi-structured interviews based on a predefined guide of open-ended core questions, together with drawing-based elicitation activities. The guide explored children’s reactions to unfamiliar foods, emotions related to tasting new foods, refusal of specific foods, fear of eating foods perceived as unsafe despite not being confirmed allergens, and parental responses to food refusal or food-related fear. The sequence of questions and the use of follow-up prompts were flexible and adapted to the child’s age, comprehension, and narrative responses [27].

The study protocol, patient information sheet, informed consent form, and data collection procedures were reviewed and approved by the Ethics Committee of the University of Naples Federico II. The study was conducted in accordance with the latest version of the Declaration of Helsinki, Good Clinical Practice standards, and applicable European and Italian regulations on personal data protection. Written informed consent was obtained from parents or legal guardians before participation. Children were informed about the study using age-appropriate language and were invited to participate voluntarily.

### 2.2. Participants

Participants were recruited among children attending the outpatient pediatric allergy clinic. Children were eligible for inclusion if they were aged 8–18 years and had a confirmed diagnosis of FA.

The food allergy diagnosis was based on clinical history and documented allergy work-up performed as part of the standard clinical care pathway at the tertiary Pediatric Allergy Unit, including skin prick testing, serum specific IgE assessment, elimination diet, and/or oral food challenge when clinically indicated, in accordance with international recommendations for FA diagnosis [2].

Children were considered eligible if they had sufficient cognitive and linguistic ability to complete the questionnaires themselves, with neutral clarification of item wording from the pediatric nurse when requested. Exclusion criteria were severe neuropsychiatric disorders, chronic non-allergic diseases, cognitive impairment preventing child self-completion of the questionnaires even with neutral clarification of item wording, and inability to understand or speak Italian, in order to ensure adequate communication and comprehension of study procedures.

### 2.3. Study Outcomes

The primary quantitative outcome was to describe the frequency of positive EDY-Q screening results for ARFID-related symptoms and the distribution of food-neophobia categories within the study sample.

Secondary outcomes included the evaluation of avoidant/restrictive eating behavior dimensions, food neophobia severity, adherence to the Mediterranean diet, and associations between eating behavior scores and diet quality. The qualitative outcome was the exploration of children’s lived experiences related to food avoidance, including emotional reactions, anticipatory fear of eating, perceived risk generalization, reassurance-seeking behaviors, coping strategies, and family responses to food-related anxiety.

### 2.4. Sampling

Given the exploratory nature of the study and the absence of sufficiently established effect-size estimates for the combined outcomes examined, no formal a priori sample-size calculation was performed. A consecutive sampling strategy was adopted, and the sample size reflected the number of eligible participants enrolled during the predefined recruitment period from August 2025 to February 2026. All eligible children attending the Pediatric Allergy Program during this period were invited to participate. Recruitment was not stratified by age, and no age quotas or additional age-based selection criteria were applied. For the qualitative component, a purposive subsample of children was selected among participants who completed the quantitative assessment, with the aim of including children with different FA profiles and varying EDY-Q scores and/or levels of food neophobia. Recruitment and preliminary coding for the qualitative component proceeded concurrently. Interviews continued until thematic saturation was reached, operationally defined as the point at which successive interviews generated no substantively new codes, subthemes, or themes and the thematic framework was considered stable by both coders. This criterion was reached after 26 interviews, at which point qualitative recruitment was discontinued. Children were invited to participate in semi-structured interviews based on open-ended core questions and to complete drawing-based elicitation activities. This approach was intended to enrich the interpretation of questionnaire findings by capturing children’s subjective experiences and emotional meanings related to food, allergy, fear, and avoidance, consistent with mixed-methods research principles [22].

### 2.5. Data Collection

Data were collected during outpatient clinical visits. After eligibility assessment and informed consent, socio-demographic and clinical data were collected using a structured form and medical chart review. Collected variables included age, sex, type of FA, allergenic food(s) involved, main clinical manifestations related to allergic reactions, and relevant allergy-related clinical history.

Then, participants completed three assessment tools: the Eating Disorders in Youth-Questionnaire (EDY-Q), the Italian Child Food Neophobia Scale (ICFNS), and the Mediterranean Diet Quality Index for Children and Adolescents (KIDMED) [23,24,25,26].

The EDY-Q was used to screen for avoidant/restrictive eating behaviors consistent with ARFID-related symptoms. The EDY-Q is a 14-item self-report questionnaire developed to assess early-onset restrictive eating disturbances in children and includes domains related to Food Avoidance Emotional Disorder, Selective Eating, Functional Dysphagia, Weight Problems, and Cognitive Distortions related to weight or body shape [23,24]. Each item is rated on a seven-point scale ranging from 0 (never) to 6 (always), and the EDY-Q total mean and domain scores therefore range from 0 to 6, with higher scores indicating greater symptom frequency or severity. A positive EDY-Q screening result for ARFID-related symptoms was defined according to the questionnaire-based criteria proposed for the instrument [23,24]. Specifically, a positive EDY-Q screening result required: (i) a score ≥4 on at least one of the three ARFID-variant items (items 2, 10, or 12); (ii) a score ≥4 on the weight-problems item (item 4); and (iii) scores <3 on both weight- and shape-concern exclusion items (items 6 and 7). No structured diagnostic interview or psychiatric assessment was performed. The EDY-Q total score was calculated as the mean of items 1–5 and 8–12, while domain scores were calculated as the mean of their corresponding items; therefore, total and domain scores ranged from 0 to 6. Such a result was interpreted as a positive screening and not as a formal clinical diagnosis of ARFID. The instrument was used for non-commercial academic research purposes in accordance with its Creative Commons license, and the original source was appropriately cited [24].

Since no formally validated Italian version of the EDY-Q was available, a study-specific Italian translation of the official English version was used. The translation retained the original item content, response options, questionnaire structure, and published scoring criteria but did not undergo formal forward–backward translation, cultural adaptation, or psychometric validation. Accordingly, EDY-Q findings were interpreted as exploratory questionnaire-based screening results.

The pica- and rumination-related items of the EDY-Q (items 13 and 14, respectively) were analyzed separately from the EDY-Q total score, domain scores, and EDY-Q screening result for ARFID-related symptoms. Item 13 assesses self-reported consumption of substances not intended as food, whereas item 14 assesses self-reported regurgitation of previously swallowed food. Both items are rated on a seven-point scale ranging from 0 (never) to 6 (always). In accordance with previous EDY-Q-based population studies [28,29], any endorsement of either behavior was defined as a score ≥1, whereas recurrent behavior was operationally defined as a score ≥4, corresponding to endorsement of the behavior at least often. Scores of 1–3 were classified as lower-frequency endorsement. The threshold of ≥4 represents an operational threshold used in previous EDY-Q research rather than a diagnostic cutoff. These classifications describe responses to individual questionnaire items and do not establish a diagnosis of pica or rumination disorder.

Food neophobia was assessed using the ICFNS, an 8-item validated child-report scale designed to evaluate children’s reluctance to try unfamiliar foods. The scale includes neophobic and neophilic items and was administered according to its original validated structure [25]. The total ICFNS score ranges from 8 to 40, with higher scores indicating greater food neophobia. Scores were categorized as low food neophobia (≤17), moderate food neophobia [18,19,20,21,22,23], and high food neophobia (≥24), according to percentile-based thresholds previously used in children assessed with the ICFNS [30].

Adherence to the Mediterranean diet was assessed using the KIDMED index, a widely used instrument for evaluating diet quality in children and adolescents. The index includes 16 items reflecting dietary behaviors consistent or inconsistent with the Mediterranean dietary pattern. The theoretical total score ranges from −4 to +12, with higher scores indicating better adherence. Scores were categorized as low (≤3), intermediate [4,5,6,7], or high (≥8) adherence [26].

The qualitative component consisted of individual, child-friendly semi-structured interviews based on a predefined guide of open-ended core questions. The interview guide included the following thematic areas: whether the child had ever been offered an unfamiliar food and how they usually reacted; how the child felt when asked to taste something never eaten before; whether there were foods the child would never eat and why; whether the child had ever thought that a food could make them feel unwell despite not being a confirmed allergen; whether the child was afraid to eat foods that should be safe; and how parents reacted when the child refused a food or reported fear of eating it. The guide included a predefined sequence of core topics, but the interviewer could adapt the order of questions and use follow-up prompts according to the child’s age, comprehension, and spontaneous narrative.

Children were also invited to complete drawing-based elicitation activities, such as drawing their favorite dish and foods they would never eat or drawing themselves while eating. Drawings were not interpreted as formal psychological tests but were used as supportive prompts to facilitate narration. After completing the drawing, children were asked to describe what they had drawn and to explain the meaning of the represented foods or eating situation.

All 26 interviews were conducted by the same pediatric nurse researcher, who had received specific training in qualitative interviewing and child-friendly communication and had no prior clinical, care, or therapeutic relationship with the participating children or their families. Interviews lasted approximately 20 min. A parent or legal caregiver was present during all 26 interviews because of the young age of the participants. Caregivers were instructed not to intervene, prompt the child, or answer questions on the child’s behalf. All questionnaires were completed by the children themselves. A pediatric nurse was available to provide neutral clarification of item wording only when requested, without suggesting responses or interpreting the items on the child’s behalf. Caregivers did not assist with questionnaire completion. In one interview, a caregiver made a spontaneous comment, which was documented as contextual information but was not included in the formal coding process.

Before data collection, the interviewer received training on the interview guide, neutral probing techniques, non-leading questioning, and the adoption of a welcoming, empathic, and non-judgmental attitude toward children’s narratives. Two pilot interviews were conducted before the start of data collection to assess the clarity, comprehensibility, timing, and flow of the interview guide. These pilot interviews were used only to refine the interview procedure and were not included in the final analysis. Brief interviewer field notes were recorded during or immediately after the interviews to document contextual and interactional elements relevant to data interpretation, including the interview setting, children’s emotional tone, pauses and hesitations, non-verbal reactions, caregiver–child interactions, and children’s engagement during drawing-based elicitation activities. Before and during data collection, the interviewer engaged in bracketing and reflexive notetaking in order to identify and temporarily set aside preconceptions, prior assumptions, and expectations that could influence question delivery, interaction with participants, or interpretation of the data [31,32].

Interviews were audio-recorded using a digital device. Recordings were transcribed verbatim by one researcher. A second researcher checked the transcripts against the audio recordings to ensure accuracy and completeness. The qualitative material was anonymized before analysis. This component was analyzed using inductive thematic analysis [27]. Interviews were conducted until thematic saturation was achieved, defined as the point at which no substantially new themes emerged from additional interviews.

### 2.6. Data Analysis

Quantitative and qualitative analyses were conducted separately and subsequently integrated during the interpretation phase, in accordance with the mixed-methods design [22].

Quantitative findings were used to describe the frequency of positive EDY-Q screening, dimensional avoidant/restrictive eating scores, food neophobia, and Mediterranean diet adherence. Qualitative findings were used to contextualize and deepen the interpretation of the quantitative results by exploring children’s lived experiences and emotional meanings related to food avoidance. Integration occurred through triangulation of quantitative and qualitative findings, comparing questionnaire results with emergent themes to identify areas of convergence, complementarity, and divergence.

All data were anonymized before analysis. The database was checked for completeness, consistency, and accuracy before statistical analysis. Questionnaires were checked for completeness before data entry. No relevant missing data were observed; therefore, complete-case analyses were performed.

### 2.7. Quantitative Analysis

Quantitative analyses were performed using IBM SPSS Statistics, version 23.0 (IBM Corporation, Armonk, NY, USA). The distribution of continuous variables was assessed using histograms, Q–Q plots, and the Shapiro–Wilk test. Normally distributed variables were summarized as mean ± standard deviation, whereas non-normally distributed variables were summarized as median and interquartile range. Categorical variables were reported as absolute frequencies and percentages.

Descriptive statistics were used to characterize the study population and questionnaire scores. EDY-Q total and subscale scores, ICFNS scores, and KIDMED scores were analyzed to describe avoidant/restrictive eating features, food neophobia, and adherence to the Mediterranean diet. Because several questionnaire scores showed non-normal distributions, Spearman’s rank correlation coefficients (ρ) were used to explore associations between EDY-Q total and subscale scores, ICFNS scores, and KIDMED scores. Given the exploratory nature of the study, correlation analyses were interpreted as hypothesis-generating, and no adjustment for multiple comparisons was applied.

Exploratory subgroup analyses were conducted according to history of anaphylaxis (yes vs. no), presence of cow’s milk protein allergy (CMPA vs. no CMPA), and number of confirmed food allergies (single vs. multiple). EDY-Q total, Selective Eating, Food Avoidance Emotional Disorder, Functional Dysphagia, ICFNS, and KIDMED scores were compared using the Mann–Whitney U test because of their non-normal distributions. Exact two-sided *p*-values were calculated. To account for multiple testing, the Benjamini–Hochberg procedure was applied across the 18 subgroup comparisons, and adjusted *p*-values are reported as q-values. These analyses were exploratory and hypothesis-generating. An exploratory hierarchical multiple linear regression analysis was performed with the KIDMED score as the dependent variable. Age, sex, and the EDY-Q Selective Eating score were entered in the first block. Age at food allergy diagnosis, expressed in months, number of confirmed food allergies, and history of anaphylaxis were entered in the second block. History of anaphylaxis was coded as 0 = no and 1 = yes, with “no” as the reference category. These clinical covariates were selected based on their clinical relevance and data availability. Given the sample size, the number of covariates was deliberately limited to reduce the risk of model overfitting. Multicollinearity was assessed using tolerance and variance inflation factor values. Linearity and homoscedasticity were evaluated through the scatterplot of standardized residuals against standardized predicted values, residual normality through the histogram and normal P–P plot, and potentially influential observations through Cook’s distance. All statistical tests were two-sided, and the level of statistical significance was set at *p* < 0.05.

### 2.8. Qualitative Analysis

Qualitative data were analyzed using an inductive thematic analysis approach, following the framework proposed by Braun and Clarke [27]. The analysis aimed to identify recurrent patterns of meaning in children’s narratives regarding food avoidance, fear of allergic reactions, perceived safety, coping strategies, and family dynamics.

The analytic process began with repeated reading of the transcripts to achieve familiarization with the data. Initial codes were then generated from meaningful segments of text. Codes were subsequently grouped into preliminary themes and subthemes based on conceptual similarity, recurrence, emotional salience, and relevance to the study aims. Themes were reviewed and refined to ensure internal coherence and clear distinction between thematic categories [27]. Preliminary coding was conducted iteratively alongside qualitative data collection, allowing emerging codes and themes to be assessed as recruitment progressed. The two coders reviewed whether successive interviews contributed substantively new codes, subthemes, or themes. Thematic saturation was considered reached when no substantively new elements emerged and both coders agreed that the thematic framework was stable and adequately represented the dataset.

Coding was conducted manually, without the use of dedicated qualitative data-analysis software. The analysis was performed independently by two researchers, who separately reviewed the transcripts and assigned initial codes to meaningful text segments. During initial coding and theme development, both researchers were blinded to participants’ EDY-Q, ICFNS, and KIDMED scores. Questionnaire scores were linked to the qualitative findings only during the subsequent mixed-methods integration phase. Disagreements in coding or theme interpretation were discussed until consensus was reached. Participant validation of the transcripts or final themes (member checking) was not performed. Drawing-based materials were not interpreted as formal psychological tests but were used as supportive elicitation tools to contextualize children’s verbal narratives and enhance understanding of their food-related experiences.

Field notes were used as contextual and reflexive material to support interpretation of the interview transcripts. They were not coded as independent data units but were consulted during theme development to clarify the emotional tone, interactional context, caregiver influence, non-verbal responses, hesitations, and drawing-related observations associated with children’s narratives.

Trustworthiness was addressed according to Lincoln and Guba’s criteria of credibility, transferability, dependability, and confirmability [32]. Credibility was supported by the use of a predefined semi-structured interview guide, child-friendly interviewing procedures, independent coding by two researchers, consensus discussions, and the selection of representative quotations. Transferability was supported by a detailed description of the study setting, participants, qualitative procedures, and thematic findings. Dependability was enhanced through documentation of the analytic process, including coding, theme development, and refinement. Confirmability was supported by interviewer bracketing, reflexive note-taking, anonymization of transcripts, and discussion of coding decisions within the research team [31,33].

Representative verbatim quotations were selected to illustrate the final themes and preserve participants’ voices. Quotations were anonymized and identified using participant codes, in accordance with confidentiality procedures and the trustworthiness criteria adopted for the qualitative component [32].

## 3. Results

### 3.1. Quantitative Findings

A total of 77 children with confirmed FA were assessed for eligibility. Seven were excluded because they met exclusion criteria: five for neuropsychiatric disorders and two for the concomitant presence of chronic non-allergic diseases.

#### 3.1.1. Demographic and Clinical Characteristics

The majority of participants were male. The median age was 8 years (IQR 8–9; range 8–9); 43 children (61.4%) were aged 8 years and 27 (38.6%) were aged 9 years. Most participants were born at term (85.7%) and by cesarean section (61.4%). Although the predefined eligibility range was 8–18 years, the participants enrolled during the study period were aged 8–9 years. The mean birth weight was 3.20 ± 0.64 kg. Breastfeeding for at least two months was reported in 58.6% of children. Maternal smoking during pregnancy was reported in 17.1% of cases, while parental smoking was present in 74.3% of families. Most children lived in an urban environment (85.7%).

FA was IgE-mediated in almost all participants (98.6%), while only one child had a non-IgE-mediated form (1.4%). The median number of allergenic foods was 2 (1–3) per child. The most frequently reported allergens were cow’s milk proteins (30.0%), egg (22.9%), peanut (20.0%), peach (18.6%), and legumes (18.6%). Tree nut allergy was reported in 12.9% of participants, lipid transfer protein sensitization in 8.6%, and fish or shellfish allergy in 5.7%.

The most frequent clinical manifestations were cutaneous symptoms, reported in 50.0% of children, followed by gastrointestinal symptoms in 25.7%, anaphylaxis in 14.3%, and respiratory symptoms in 10.0%.

Detailed demographic and clinical characteristics of the study population are reported in Table 1.

#### 3.1.2. EDY-Q Findings and Screening Results

Eating behavior outcomes are summarized in Table 2. The median EDY-Q total score was 1.05 (25th–75th percentile: 0.40–2.40). Based on the EDY-Q screening criteria, 3 children (4.3%) had a positive screening result for ARFID-related symptoms, whereas 67 children (95.7%) had a negative screening result.

Among the EDY-Q domains, Selective Eating showed the highest median score [1.50 (0.67–4.00)], followed by FAED [1.00 (0.00–2.00)]. Functional Dysphagia had a median score of 0.00 (0.00–2.00). Weight Problems and Cognitive Distortions related to weight or body shape both showed marked floor effects, with median scores of 0.00 (0.00–1.00) and 0.00 (0.00–0.00), respectively (Table 2).

Rumination-related behavior was assessed descriptively using EDY-Q item 14. No endorsement was reported by 49 children (70.0%), whereas 21 children (30.0%) endorsed the item at any level (score ≥1). Of these, 15 children (21.4% of the total sample) reported lower-frequency endorsement (scores 1–3), while 6 children (8.6%) reached the operational threshold for recurrent rumination-related behavior (score ≥4). As this finding was based on a single questionnaire item without a structured clinical assessment, it should not be interpreted as indicating a diagnosis of rumination disorder. Pica-related behavior was not endorsed by 67 children (95.7%), while three children (4.3%) reported lower-frequency endorsement (scores 1–3). No participant reached the operational threshold of ≥4 for recurrent pica-related behavior.

#### 3.1.3. Food Neophobia

The ICFNS showed a mean score of 22.76 ± 2.30 (Table 2). Most children presented moderate food neophobia (*n* = 45; 64.3%), while 24 children (34.3%) showed high food neophobia. Only one participant (1.4%) had low food neophobia. These findings describe the distribution of food neophobia categories within this tertiary-care sample and should not be interpreted as population prevalence estimates.

#### 3.1.4. Mediterranean Diet Adherence

The median KIDMED score was 6.00 (25th–75th percentile: 5.00–8.00). Most participants showed intermediate adherence, while approximately one third showed high adherence. Low adherence was observed in a minority of children (Table 2).

#### 3.1.5. Correlation and Regression Analyses

Spearman’s rank-order correlation analysis was performed to explore associations between the EDY-Q total score, EDY-Q subscales, ICFNS score, and KIDMED score. The main correlations of clinical interest are reported in Table 3.

The EDY-Q total score showed strong positive correlations with Selective Eating (ρ = 0.912; *p* < 0.001), FAED (ρ = 0.826; *p* < 0.001), and Functional Dysphagia (ρ = 0.754; *p* < 0.001). A moderate positive correlation was also observed with Weight Problems (ρ = 0.488; *p* < 0.001), whereas Cognitive Distortions were not significantly correlated with the EDY-Q total score (ρ = 0.071; *p* = 0.558).

Among the EDY-Q subscales, Selective Eating was positively correlated with FAED (ρ = 0.678; *p* < 0.001), Functional Dysphagia (ρ = 0.667; *p* < 0.001), and Weight Problems (ρ = 0.415; *p* < 0.001). Functional Dysphagia was also positively correlated with FAED (ρ = 0.505; *p* < 0.001) and Weight Problems (ρ = 0.309; *p* = 0.009).

ICFNS score was not significantly correlated with the EDY-Q total score (ρ = 0.040; *p* = 0.745). A weak positive correlation was observed between ICFNS score and Cognitive Distortions (ρ = 0.258; *p* = 0.031); however, this finding should be interpreted cautiously because of the marked floor effect in this EDY-Q domain and the exploratory nature of the analyses.

KIDMED score was negatively correlated with the EDY-Q total score (ρ = −0.302; *p* = 0.011), Selective Eating (ρ = −0.356; *p* = 0.002), and Functional Dysphagia (ρ = −0.238; *p* = 0.048). The inverse correlation between KIDMED and FAED did not reach statistical significance (ρ = −0.231; *p* = 0.054). No significant correlation was observed between KIDMED and ICFNS (ρ = 0.160; *p* = 0.185).

In the hierarchical multiple linear regression, the initial model including age, sex, and the EDY-Q Selective Eating score was statistically significant and explained 12.5% of the variance in KIDMED scores (R^2^ = 0.125, adjusted R^2^ = 0.086; F(3,66) = 3.151, *p* = 0.031). After age at FA diagnosis, number of confirmed food allergies, and history of anaphylaxis were added, the EDY-Q Selective Eating score retained a significant inverse coefficient with KIDMED (B = −0.455, SE = 0.143, β = −0.386, 95% CI −0.740 to −0.169, *p* = 0.002). The additional clinical covariates did not significantly improve model fit (ΔR^2^ = 0.034, ΔF(3, 63) = 0.847, *p* = 0.473), and none was independently associated with KIDMED. The fully adjusted model explained 15.9% of the variance but did not reach overall statistical significance (F(6, 63) = 1.988, *p* = 0.081). Accordingly, the fully adjusted findings should be interpreted as exploratory.

#### 3.1.6. Exploratory Subgroup Analyses

Exploratory subgroup analyses were conducted according to history of anaphylaxis, CMPA status, and single versus multiple confirmed food allergies. No significant differences in EDY-Q total, Selective Eating, FAED, Functional Dysphagia, ICFNS, or KIDMED scores were observed between children with a single confirmed food allergy (*n* = 30) and those with multiple confirmed food allergies (*n* = 40); none of these comparisons approached statistical significance after Benjamini–Hochberg correction (all q ≥ 0.491).

Children with a history of anaphylaxis (*n* = 10) showed nominally higher FAED and Selective Eating scores than children without a history of anaphylaxis (*n* = 60; exact *p* = 0.041 and *p* = 0.045, respectively). However, neither difference remained statistically significant after correction for multiple comparisons (q = 0.135 for both). The difference in EDY-Q total score also did not reach statistical significance (exact *p* = 0.053; q = 0.136). None of the subgroup differences according to anaphylaxis history remained significant after Benjamini–Hochberg correction.

Children with CMPA (*n* = 21) showed lower EDY-Q total scores than children without CMPA (median 0.40 [IQR 0.10–1.00] vs. 1.70 [0.60–2.55]; exact *p* = 0.001; q = 0.018). They also showed lower FAED scores (0.00 [0.00–1.00] vs. 1.33 [0.17–2.67]; exact *p* = 0.003; q = 0.018) and Selective Eating scores (0.67 [0.00–1.83] vs. 2.33 [1.00–4.00]; exact *p* = 0.003; q = 0.018). The nominal difference in Functional Dysphagia did not remain statistically significant after correction (exact *p* = 0.027; q = 0.122). No differences in ICFNS or KIDMED scores were observed according to CMPA status. Given the small subgroup sizes and post hoc exploratory nature of these comparisons, the findings should be considered hypothesis-generating.

### 3.2. Qualitative Findings

A qualitative subsample of 26 children with IgE-mediated FA and varying EDY-Q scores and/or levels of food neophobia, including 17 boys (65.4%) and 9 girls (34.6%), participated in the semi-structured interviews and drawing-based activities.

A parent or legal caregiver was present during all 26 interviews. Caregivers were instructed not to intervene or answer questions on behalf of the child. Thematic analysis identified a structured pattern of emotional, behavioral, and relational responses to food and mealtimes. Five main themes were identified: (1) anticipatory fear of food and tasting; (2) emotional memory of allergic reactions and generalization of risk; (3) selective avoidance and food rigidity; (4) constant reassurance-seeking and decision-making dependence; and (5) ambivalent parental dynamics. Within these themes, recurrent subthemes were identified, including perceived food threat, somatic anticipation, uncertainty about hidden ingredients, emotional memory of previous reactions, sensory generalization of risk, refusal of unfamiliar foods, ingredient-related uncertainty, caregiver-mediated safety checking, limited food autonomy, and inconsistent parental responses. Subthemes helped to clarify how food avoidance was expressed across different levels, including emotional anticipation, bodily sensations, sensory generalization, uncertainty about ingredients, caregiver-mediated safety checking, and family responses to refusal. Field notes provided additional contextual information supporting the interpretation of the qualitative findings. Most interviews were conducted in a private outpatient room without overlap with other patients, allowing sufficient time to establish rapport and complete the interview without interruption. In several interviews, children initially showed hesitation or mild embarrassment when answering open-ended questions, whereas most appeared more relaxed and engaged during drawing-based activities. Caregiver-related field-note observations were treated solely as contextual information and were not coded as independent qualitative data. Because a caregiver was present during all interviews, the possible influence of caregiver presence on children’s responses could not be assessed empirically. 

During drawing activities, most children appeared calm and were able to describe the foods represented, often explaining whether the food was avoided because of allergy, dislike, or perceived danger. Themes, subthemes, descriptive thematic summary and representative quotations are summarized in Table 4.

#### 3.2.1. Theme 1. Anticipatory Fear of Food and Tasting

Food, particularly when unfamiliar or not fully known, was frequently perceived as a potential threat. Several children described food-related fear as immediate, bodily, and closely connected to the possibility of feeling unwell.

One child stated: “I feel like vomiting” (ID1, male, IgE-mediated allergy to milk, egg, and apple). Another child reported: “I am afraid I will have an allergic reaction” (ID4, male, IgE-mediated allergy to peach, tree nuts, and strawberry; history of anaphylaxis). A third participant described a more somatic fear: “I feel short of breath in my throat. I do not want to eat anything” (ID50, male, IgE-mediated allergy to strawberry and peach). Similarly, another child stated: “I am always afraid of what I eat because I do not know what is inside” (ID62, female, IgE-mediated allergy to fish and shellfish).

Across these accounts, unfamiliar or uncertain foods were frequently described in terms of perceived danger and anticipated bodily symptoms.

#### 3.2.2. Theme 2. Emotional Memory of Allergic Reactions and Generalization of Risk

Participants linked previous allergic reactions with subsequent food-related fear and described avoidance based on similarities in food color, texture, or appearance.

One child reported: “I felt sick, so now I am afraid” (ID8, male, IgE-mediated allergy to red meat; history of anaphylaxis). Another participant stated: “I had a bad reaction to tuna, and I was afraid” (ID17, female, IgE-mediated allergy to fish). In the same case, the caregiver reported: “The first time we realized she was allergic, she was eating sole and she was traumatized” (ID17, female, IgE-mediated allergy to fish).

The memory of the allergic reaction appeared to acquire an emotional meaning that went beyond the clinical event itself. In several cases, fear generalized to foods with similar sensory characteristics. One child stated: “Anything yellow scares me because maybe it is banana” (ID3, male, IgE-mediated allergy to banana). Another reported: “Apricot has skin like peach” (ID43, male, IgE-mediated allergy to peach; history of anaphylaxis). A third child described: “Many things have the same texture as meat, and then I feel sick” (ID65, female, IgE-mediated allergy to red meat and milk).

These accounts indicate that perceived risk was no longer limited to the documented allergen but extended to sensory features that became symbolic warning signals.

#### 3.2.3. Theme 3. Selective Avoidance and Food Rigidity

Many children described a restricted food repertoire and a stable tendency to refuse new or uncertain foods. One child stated: “I never eat what I do not know” (ID1, male, IgE-mediated allergy to milk, egg, and apple). Another participant reported: “I do not want to eat almost anything because I do not like things I do not know” (ID66, male, IgE-mediated allergy to soy, peas, lupins, and hazelnuts). A further child said: “I never eat it because I do not know what they put inside” (ID70, female, IgE-mediated allergy to peanut).

Avoidance also extended to sensory characteristics such as color, consistency, or visual appearance. One child reported: “I do not want to eat spinach because it looks ugly on the plate” (ID69, female, IgE-mediated allergy to legumes; history of anaphylaxis), while another stated: “I do not want to eat rice with spinach, pasta with pumpkin, or zucchini because I do not like the texture” (ID45, female, IgE-mediated allergy to tree nuts, soy, legumes, and lipid transfer proteins).

In these accounts, refusal was described not only in relation to confirmed allergens but also in the context of uncertainty, sensory characteristics, and food-related anxiety.

#### 3.2.4. Theme 4. Constant Reassurance-Seeking and Decision-Making Dependence

The evaluation of food safety was frequently delegated to caregivers. Several children described a persistent need for confirmation before eating uncertain or unfamiliar foods. One child stated: “I always ask my mother if I can eat it” (ID29, male, IgE-mediated allergy to grape; history of anaphylaxis). Another participant reported: “I ask my mother or father whether I can eat it” (ID49, female, IgE-mediated allergy to peanut and peas). A third participant stated: “I always ask if there is garlic” (ID58, male, IgE-mediated allergy to garlic; history of anaphylaxis).

These accounts described frequent reliance on caregivers for ingredient checking and confirmation of food safety.

#### 3.2.5. Theme 5. Ambivalent Parental Dynamics

Parental responses appeared to oscillate between protection, verbal reassurance, and pressure to taste foods. One child stated: “My mother insists, my father does not” (ID1, male, IgE-mediated allergy to milk, egg, and apple). Another reported: “Dad says that if I do not want it, it is okay, but mom always says I have to try” (ID69, female, IgE-mediated allergy to legumes; history of anaphylaxis). A further child stated: “If I keep eating nothing, I will stay small forever” (ID50, male, IgE-mediated allergy to strawberry and peach). Children described different parental responses to food refusal, ranging from reassurance and acceptance to repeated encouragement or pressure to taste.

### 3.3. Drawing-Based Activities

Drawing-based activities supported and enriched the themes identified through interviews. In several cases, children represented the feared or allergenic food rather than the preferred one. For example, one child drew an apple and explained: “Because I am allergic” (ID1, male, IgE-mediated allergy to milk, egg, and apple). Another child drew a banana and stated: “I do not even want to see yellow things” (ID3, male, IgE-mediated allergy to banana).

A female participant drew a sole and explained: “It is a sea sole with the scales—the things that bother you in your mouth when you eat” (ID17, female, IgE-mediated allergy to fish).

In other cases, drawings visually separated liked and disliked foods. Drawings frequently depicted allergenic, feared, disliked, or preferred foods and were used only as elicitation prompts for children’s verbal accounts; they were not interpreted as formal psychological assessments.

### 3.4. Mixed-Methods Integration

Quantitative and qualitative findings were integrated after the two components had been analyzed independently. A formal Joint Display was developed to align the principal quantitative findings with the corresponding qualitative themes and representative quotations and to identify areas of convergence, complementarity, and divergence (Table 5). Because the qualitative subsample was purposively selected, qualitative findings were used to deepen the interpretation of the quantitative results and not to estimate the frequency of experiences in the overall study population.

Integration of the quantitative and qualitative findings revealed convergence, complementarity, and divergence. The qualitative themes of anticipatory fear, generalization of perceived risk, and selective food avoidance were consistent with the EDY-Q findings concerning avoidant/restrictive eating features and contextualized how these experiences were described by children. Narratives concerning restricted food repertoires and ingredient-related uncertainty also complemented the inverse correlations between KIDMED and EDY-Q total score, Selective Eating, and Functional Dysphagia, without establishing directionality. At the same time, qualitative accounts described food-related distress and caregiver-dependent reassurance among children who did not necessarily have a positive EDY-Q screening result. This represented complementarity between the two methods and should not be interpreted as evidence of additional or undiagnosed ARFID cases. A divergence concerned food neophobia: despite the predominance of moderate and high ICFNS categories within the study sample, ICFNS scores were not significantly correlated with the EDY-Q total score or its main domain scores. This finding is consistent with food neophobia and EDY-Q-assessed avoidant/restrictive eating features representing partially overlapping but distinguishable constructs. Finally, the qualitative themes concerning emotional memory, sensory generalization of perceived risk, and caregiver reassurance generated hypotheses that were not directly tested by the quantitative component. These integrated findings should therefore be considered hypothesis-generating rather than evidence of temporal, causal, or mechanistic relationships.

**Figure 1:**
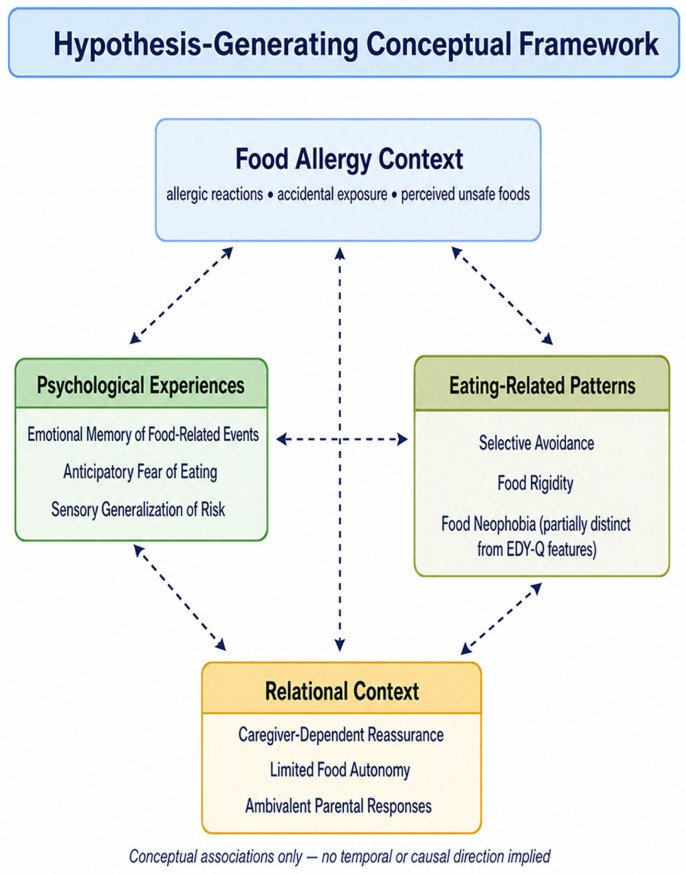
Hypothesis-generating conceptual framework of food-related psychological experiences, eating-related patterns, and relational factors in the study sample. The framework summarizes selected qualitative themes interpreted alongside the quantitative findings; the full mixed-methods integration is presented in Table 5. Dashed bidirectional connectors denote conceptual co-occurrence or possible association only and do not imply temporal ordering, mediation, mechanism, or causality.

Figure 1 provides a hypothesis-generating conceptual synthesis of selected qualitative themes interpreted alongside the quantitative findings. It is complementary to the full mixed-methods Joint Display presented in Table 5 and does not represent a causal pathway.

## 4. Discussion

This mixed-methods study provides a multidimensional assessment of EDY-Q–assessed ARFID-related symptoms, food neophobia, diet quality, and food-related psychological experiences in children with confirmed FA. Within this tertiary-care sample, only 3 of 70 participants (4.3%) had a positive EDY-Q screening result for ARFID-related symptoms, whereas most participants were classified within the moderate food-neophobia category and approximately one third within the high category. Selective Eating showed the highest median EDY-Q domain score and was associated with lower adherence to the Mediterranean diet. The qualitative component added contextual depth to these findings: interviewed children described anticipatory fear, emotional memory of allergic reactions, sensory generalization of perceived risk, selective avoidance, caregiver-dependent reassurance, and ambivalent parental responses in relation to their eating experiences.

The contrast between the low frequency of positive EDY-Q screening results and the broader occurrence of food neophobia and food-related avoidance is a central finding of the study. These findings should not be considered inconsistent, because the assessed phenomena are related but not equivalent. A positive EDY-Q screening result reflects fulfillment of a relatively specific set of questionnaire-based criteria for ARFID-related symptoms [23,24] but does not establish a clinical diagnosis of ARFID [13,14]. Food neophobia, by contrast, represents a dimensional reluctance to try unfamiliar foods and does not necessarily involve nutritional, growth-related, or psychosocial impairment [16,17,18]. Similarly, the qualitative accounts of food-related fear, uncertainty, avoidance, and reassurance-seeking should not be interpreted as evidence of additional ARFID cases. Moreover, because the qualitative subsample was purposively selected, the recurrence of these themes cannot be used to estimate their frequency in the full cohort. In this sample, positive EDY-Q screening results were less frequent than dimensional avoidant/restrictive eating features and moderate or high food-neophobia categories. The cross-sectional data do not establish whether these phenomena form a single severity continuum.

The sample-specific frequency of positive EDY-Q screening results observed in this study is difficult to compare directly with previous findings in FA populations. Earlier studies have predominantly examined broadly defined feeding difficulties or disordered eating behaviors, whereas ARFID-specific publications in FA have often involved clinical reports or highly selected cases rather than standardized screening in consecutively recruited pediatric allergy samples [9,11,12,34]. The systematic review by Ciciulla et al. documented substantial heterogeneity in study populations, definitions, assessment instruments, and diagnostic procedures, precluding a robust pooled estimate of ARFID in individuals with FA [9]. Clinical reports have identified ARFID as a potentially treatable complication of FA but have not established its frequency in the general pediatric FA population [11,12]. Similarly, the EAACI Task Force Report emphasized that feeding difficulties in children with FA are heterogeneous, variably defined, and likely under-recognized in routine allergy care [34]. Consequently, the present 4.3% estimate should be interpreted as a sample-specific questionnaire-based screening frequency and should not be considered higher or lower than previously reported estimates without accounting for differences in recruitment setting, age, exclusion criteria, assessment instruments, and diagnostic procedures.

Two very recent controlled studies provide further context for these findings. In the population-based HealthNuts cohort, Ciciulla et al. administered the EDY-Q to 951 10-year-old children, including 102 with current FA. Using an updated operational definition that removed the original subjective-underweight requirement, possible ARFID was identified in 23% of children with current FA and 21% of those without FA. However, when the original EDY-Q scoring criteria, which retained the subjective-underweight requirement, were applied, the frequency in the overall cohort was 5%, which is close to the 4.3% observed in our study [35]. The authors also noted that the EDY-Q does not directly capture fear of allergic reactions, potentially limiting its sensitivity in FA populations. In a prospective controlled study of 117 children with physician-diagnosed FA and 117 age- and sex-matched healthy controls, Karaca Şahin et al. found higher parent-reported NIAS total scores in the FA group, primarily driven by fear-related and picky-eating dimensions, whereas appetite scores did not differ [36]. Fear-related scores were also higher in children with a history of urticaria or anaphylaxis. Together, these recent findings support the distinction between categorical screening results and dimensional eating-related difficulties and are consistent with the prominence of Selective Eating and fear-related qualitative themes in our sample.

The distribution of EDY-Q subscale scores further clarifies the clinical profile of eating difficulties in this cohort. Selective Eating showed the highest median score, followed by Food Avoidance Emotional Disorder and Functional Dysphagia, whereas weight-related problems and cognitive distortions concerning weight or body shape were low. This pattern supports the interpretation that eating difficulties in children with FA are not primarily driven by weight- or shape-related concerns, but rather by selectivity, fear, sensory sensitivity, and perceived risk. This is coherent with the ARFID construct, in which restriction may be driven by sensory sensitivity, low interest in food, or fear of aversive consequences such as choking, vomiting, gastrointestinal discomfort, or allergic reactions [13,15]. In children with FA, the fear-of-aversive-consequences profile may be particularly relevant because allergic reactions are not imagined risks but possible clinical events that children and families are explicitly taught to prevent.

Exploratory subgroup analyses suggested lower EDY-Q total, FAED, and Selective Eating scores among children with CMPA. This finding should not be interpreted as indicating a protective effect of CMPA. It may reflect differences in allergy history, duration of dietary management, familiarity with established safe alternatives, clinical education, or other unmeasured characteristics. Because these were unadjusted cross-sectional comparisons, residual confounding cannot be excluded. Children with previous anaphylaxis showed nominally higher emotional food avoidance and selective eating scores, a pattern potentially consistent with greater food-related threat perception; however, these differences did not remain significant after false discovery rate correction and were based on only ten children with anaphylaxis. These subgroup findings should therefore be considered hypothesis-generating.

The endorsement of rumination-related behavior warrants specific consideration. Although 30.0% of participants endorsed EDY-Q item 14 at any level, most responses were below the operational cutoff for recurrent behavior: 21.4% of the total sample reported lower-frequency endorsement, while 8.6% scored ≥4. These frequencies appear higher than those reported in previous population-based pediatric studies using the same EDY-Q item [28,29]. In contrast, pica-related endorsement was uncommon and limited to lower-frequency responses, with no participant reaching the operational threshold for recurrent behavior. As this finding was based on a single EDY-Q item, it should not be interpreted as evidence of clinically diagnosed pica. However, the present findings were obtained in a small tertiary-care food allergy sample without a healthy comparison group and should therefore be interpreted cautiously. In children with food allergy, endorsement of this item may reflect heterogeneous experiences, including gastrointestinal discomfort, gastroesophageal reflux-like symptoms, nausea, swallowing difficulties, fear of adverse food reactions, or heightened attention to postprandial bodily sensations. Children may also have interpreted the item as referring to vomiting or reflux rather than effortless recurrent regurgitation. Because EDY-Q item 14 does not assess symptom duration, medical or gastrointestinal exclusions, associated impairment, or the full diagnostic features of rumination disorder, the present results should be regarded as questionnaire-based signals warranting further clinical assessment rather than evidence of a clinical disorder.

The distribution of food neophobia categories represents another noteworthy finding. In the present sample, most children were classified as having moderate food neophobia, while approximately one third were classified within the high food neophobia category. However, because the study did not include a healthy control group or another clinical comparison group, these proportions cannot establish whether food neophobia was more frequent in this cohort than in the general pediatric population or among children with other chronic conditions. Food neophobia is commonly defined as reluctance or refusal to try unfamiliar foods and may be considered developmentally expected in early childhood; however, persistent or severe forms may reduce dietary variety and compromise diet quality [16,17,18]. In children with FA, this phenomenon may have a specific clinical meaning because unfamiliar foods may be perceived not only as novel, but also as potentially unsafe. This interpretation is supported by recent evidence showing that FA may be associated with altered taste perception, food neophobia, and reduced flexibility toward foods [10,19,20].

Interestingly, food neophobia was not significantly correlated with the EDY-Q total score or with the main EDY-Q subscales. This suggests that food neophobia and ARFID-related symptoms, although clinically overlapping, may represent partially distinct dimensions of eating behavior in children with FA. ARFID-related symptoms assessed through the EDY-Q appeared more closely connected to selective eating, emotional food avoidance, functional dysphagia, and fear-related eating difficulties. By contrast, food neophobia may reflect a broader reluctance toward unfamiliar foods that is not necessarily associated with the full avoidant/restrictive profile captured by EDY-Q. This distinction is clinically relevant because screening for ARFID symptoms alone may underestimate meaningful eating-related difficulties in children with FA. Conversely, assessing food neophobia without exploring fear, functional impairment, nutritional consequences, and psychosocial burden may overlook children who are progressing toward more clinically significant avoidant/restrictive patterns. The weak association observed between food neophobia and cognitive distortions related to weight or body shape should not be overinterpreted, given the very low scores in this EDY-Q domain and the exploratory nature of the correlation analyses.

A further key finding concerns the relationship between eating behavior and diet quality. KIDMED score was negatively correlated with the EDY-Q total score, Selective Eating, and Functional Dysphagia. Among the individual EDY-Q domains, Selective Eating showed the strongest inverse correlation with Mediterranean diet adherence, supporting the possibility that greater food selectivity may be associated with reduced dietary variety and poorer overall diet quality. A weak inverse correlation was also observed between KIDMED and Functional Dysphagia; however, given its small magnitude, borderline statistical significance, and the exploratory nature of the analyses without correction for multiple comparisons, this finding should be interpreted cautiously and requires confirmation in larger samples. The inverse correlation between KIDMED and FAED did not reach statistical significance, although its direction was consistent with greater emotional food avoidance being associated with poorer diet quality. The Mediterranean dietary pattern is characterized by variety and regular consumption of nutrient-rich foods, including fruits, vegetables, legumes, cereals, fish, and olive oil [26]. Selective eating and swallowing-related concerns may therefore be associated with reduced access to several components of a balanced dietary pattern, particularly when FA already imposes medically necessary restrictions. This interpretation is consistent with nutritional literature emphasizing that elimination diets are not risk-free and that dietary management of FA should aim not only to prevent reactions but also to preserve nutritional adequacy, dietary diversity, growth, and long-term health [37,38]. Notably, KIDMED was not significantly correlated with ICFNS, supporting the interpretation that diet quality may be more closely associated with specific avoidant/restrictive eating dimensions than with food neophobia per se in this cohort.

The qualitative findings substantially enriched the interpretation of the quantitative data. Although only a minority of children screened positive for ARFID symptoms, interviews revealed a broader and clinically meaningful experience of food-related fear. Several children described food-related fear through bodily sensations such as nausea, throat discomfort, shortness of breath, or fear of an allergic reaction. These findings suggest that food-related anxiety in children with FA may be experienced not only cognitively but also somatically, reinforcing avoidance behaviors. This aligns with previous work showing that FA-specific anxiety can contribute to maladaptive coping, excessive restriction, and impaired quality of life when it exceeds adaptive vigilance [6,21].

The theme of emotional memory and generalization of risk is particularly relevant. In our qualitative findings, memory of an allergic reaction emerged as an emotionally salient experience, while generalization of risk to sensory characteristics such as color, texture, appearance, or similarity to the allergen was observed in several narratives. This pattern is consistent with the possibility that medically appropriate allergen avoidance may coexist with avoidance of tolerated foods perceived as dangerous. Children’s narratives suggested that FA was experienced not only as a medical condition requiring avoidance but also as an emotionally salient context associated with subsequent food-related expectations. However, the cross-sectional design does not establish the temporal direction of these experiences. This interpretation is consistent with the conceptual model proposed by Proctor et al., according to which medically required food avoidance, allergic reactions, uncertainty, and fear of adverse consequences may increase vulnerability to fear-based ARFID pathways in pediatric FA [11].

Selective avoidance and food rigidity were also central in the qualitative narratives. Children often described refusing unknown foods, foods with uncertain ingredients, or foods with unpleasant sensory features. This qualitative pattern mirrors the quantitative finding that Selective Eating was the highest EDY-Q domain and the EDY-Q subscale most clearly associated with poorer KIDMED scores. Taken together, these findings indicate that selective eating was frequently described in the context of uncertainty and perceived food-related safety. However, when this strategy extends beyond confirmed allergens, it may contribute to nutritional narrowing and reduced dietary flexibility. This distinction between medically indicated avoidance and maladaptive over-restriction is clinically central in pediatric FA [10,11,12].

Another important qualitative finding was the frequent dependence on caregiver reassurance. Many children delegated food safety decisions to caregivers rather than describing autonomous criteria for evaluating risk. While parental supervision is essential in pediatric FA, excessive reassurance-seeking may maintain anxiety and delay the development of age-appropriate food autonomy. This is particularly relevant during school age, when children gradually begin to eat outside the home and must learn to manage FA safely but flexibly. Previous literature has shown that parental anxiety, maternal distress, and overprotection may influence children’s psychosocial functioning, quality of life, and FA-related anxiety [39,40]. Therefore, caregiver reassurance should be understood as a necessary safety behavior when proportionate, but as a potential maintaining factor when it becomes repetitive, excessive, or substitutes for the child’s gradual acquisition of self-management skills.

The theme of ambivalent parental dynamics further highlights the complexity of family management. Children described parental responses that oscillated between reassurance, pressure to taste, and acceptance of refusal. Such inconsistency may unintentionally reinforce uncertainty around food. Excessive pressure may increase distress and resistance, whereas excessive accommodation may maintain avoidance. This pattern is consistent with broader literature on parental feeding practices, which suggests that parent–child feeding interactions are bidirectional and that pressure-based feeding may contribute to negative mealtime experiences and persistent refusal in vulnerable children [41,42]. In children with FA, this issue is even more delicate because caregivers must simultaneously support safety, dietary expansion, and emotional regulation.

Taken together, the quantitative and qualitative findings are consistent with a hypothesis-generating conceptual framework in which food-related threat experiences, emotional memory, anticipatory fear, sensory generalization of perceived risk, selective avoidance, and caregiver reassurance are interrelated. The cross-sectional design does not establish the temporal order or causal relationships among these experiences. Avoidant/restrictive eating in children with FA may therefore be understood as occurring at the intersection of medical risk, dietary management, emotional experiences, family responses, and child development. This interpretation is consistent with the growing literature emphasizing integrated approaches to FA that address medical safety, nutritional adequacy, psychosocial burden, and quality of life.

Alongside vulnerability, the qualitative findings suggested possible expressions of resilience and adaptive coping. Food-related vigilance was not invariably accompanied by rigid or maladaptive restriction. Some children remained curious about unfamiliar foods, were willing to taste them after appropriate safety checking, distinguished confirmed allergens from tolerated foods, and accepted calm reassurance from caregivers. In this context, asking a caregiver to verify ingredients may represent a developmentally appropriate safety strategy rather than, in itself, maladaptive dependence, particularly in children aged 8–9 years. Accurate allergy knowledge, confidence in identifying safe foods, calm family communication, predictable safety routines, and the gradual development of food-related autonomy may therefore represent potential protective factors. However, resilience and coping were not assessed using dedicated standardized measures; these qualitative observations should consequently be considered hypothesis-generating and warrant evaluation in future longitudinal studies. These findings suggest several potential clinical implications. Pediatric allergists may benefit from incorporating brief, age-appropriate questions about persistent food refusal, fear of eating or tasting, avoidance extending beyond confirmed allergens, dependence on caregiver reassurance, and possible nutritional or psychosocial consequences. When clinically relevant concerns are identified, standardized screening instruments may support, but should not replace, a comprehensive clinical assessment. A positive screening result or evidence of significant functional impairment may warrant multidisciplinary evaluation involving the pediatric allergist, pediatrician, dietitian, and, where appropriate, a psychologist or child mental-health professional. Psychological support may be particularly relevant when fear of adverse reactions, avoidance of tolerated foods, mealtime distress, or reassurance-seeking behaviors persist despite appropriate allergy education. Family education may also benefit from addressing the distinction between necessary allergen avoidance and excessive restriction, while supporting safe exposure to tolerated foods and the child’s progressive autonomy. Nevertheless, the present exploratory findings do not establish the effectiveness or cost-effectiveness of universal screening or routine psychological assessment for all children with FA. In this perspective, validated tools for FA-associated anxiety and coping may be useful to identify families who need additional psychosocial support [43].

This study has several strengths. The mixed-methods design allowed questionnaire-based assessment of ARFID-related symptoms, food neophobia, and diet quality to be integrated with children’s own narratives. This approach provided a more nuanced understanding of eating difficulties than questionnaire data alone. The study also focused on an underexplored clinical population and used previously published, standardized instruments for quantitative assessment. Furthermore, the qualitative component allowed the identification of experiences and potentially relevant psychological and relational processes that may not be captured by standard clinical assessment, such as sensory generalization of perceived risk, emotional memory of allergic reactions, and caregiver-dependent reassurance.

Several limitations should be acknowledged. First, the cross-sectional design does not permit causal inferences or establish the temporal sequence among FA-related experiences, food neophobia, ARFID-related symptoms, and diet quality. Second, consecutive recruitment at a single tertiary pediatric allergy center may have introduced selection and referral bias. Children attending tertiary services may have more complex allergy profiles, more severe previous reactions, multiple food exclusions, or greater food-related concerns than children managed in community settings. Conversely, the exclusion criteria may have omitted children with relevant neuropsychiatric or chronic comorbidities. Together with the restricted age range of 8–9 years, these factors limit generalizability and may have influenced the sample-specific frequency estimates. Third, the relatively small sample size limited subgroup analyses and the number of covariates that could be included in multivariable models. Fourth, the EDY-Q is a screening questionnaire and cannot establish a formal ARFID diagnosis. No structured psychiatric diagnostic interview was performed, and the study-specific Italian translation of the EDY-Q has not undergone formal linguistic and psychometric validation. Fifth, questionnaire findings were based on child self-report and may therefore be affected by recall, comprehension, and social-desirability bias. Although neutral clarification was provided by a pediatric nurse only when requested, responses were not independently verified. Sixth, the absence of a healthy or clinical comparison group and of an independent external validation cohort prevents determination of whether the observed frequencies differ from those of the general pediatric population and limits external validity. Seventh, other potentially relevant variables—including gastrointestinal symptoms, reaction severity, number of foods actually avoided, FA-specific and parental anxiety, socioeconomic characteristics, parental education, and detailed dietary intake—were not fully assessed or included in the regression model; therefore, residual confounding cannot be excluded. Eighth, KIDMED provides an overall indicator of Mediterranean diet adherence but does not replace detailed dietary assessment, nutrient analysis, or longitudinal growth monitoring. Finally, all caregivers were present during the qualitative interviews, which may have influenced children’s responses and introduced social-desirability bias. Member checking was not performed, and drawing-based activities were used only as supportive elicitation tools rather than formal psychological assessments.

Future studies should include larger multicenter cohorts, appropriately matched healthy or clinical comparison groups, longitudinal designs, structured diagnostic assessment for ARFID, validated measures of FA-specific anxiety and parental anxiety, and detailed dietary and growth assessments. Where developmentally and ethically appropriate, future qualitative studies should consider conducting child-only interviews or systematically comparing interviews conducted with and without caregiver presence. Such studies would help clarify whether early food-related fear, sensory generalization of risk, selective avoidance, and reassurance-seeking behaviors predict persistent restrictive eating patterns, reduced dietary variety, or poorer nutritional outcomes over time.

## 5. Conclusions

In conclusion, within this tertiary-care sample of children with FA, a positive EDY-Q screening result for ARFID-related symptoms was observed in a minority of participants, whereas broader food-related difficulties—including food neophobia, selective eating, anticipatory fear, sensory generalization of perceived risk, and reassurance-seeking—were observed across questionnaire findings and qualitatively described within the purposive interview subsample. Selective eating was associated with lower adherence to the Mediterranean diet, although the cross-sectional design does not establish a causal relationship. These findings suggest that pediatric FA care may benefit from greater attention to eating behavior and from consideration of whether food avoidance extends beyond confirmed allergens. Children presenting with persistent fear, avoidance of tolerated foods, nutritional consequences, or psychosocial impairment may benefit from targeted multidisciplinary assessment. Larger, controlled, and appropriately powered studies using validated diagnostic procedures are needed before recommending universal EDY-Q screening for ARFID-related symptoms or routine psychological assessment for all children with FA.

## Figures and Tables

**Table 1:** Demographic and clinical characteristics of the study population (*n* = 70).

Variable	Descriptive Statistics
Sex	Male: 41 (58.6%); Female: 29 (41.4%)
Age at recruitment (years)	8 (8–9); range 8–9
Weight (kg)	28.1 (24.1–31.7)
Height (cm)	124.6 (120.1–125.6)
BMI (kg/m^2^)	18.13 ± 1.92
Birth weight (g)	3195.21 ± 642.79
Age at weaning (months)	6 (5–6)
Age at FA diagnosis (months)	24 (12–36)
Duration of FA (months)	78 (72–84)
Gestational age	Term: 60 (85.7%); Preterm: 10 (14.3%)
Mode of delivery	Cesarean: 43 (61.4%); Vaginal: 27 (38.6%)
Breastfeeding ≥2 months	41 (58.6%)
Maternal smoking during pregnancy	12 (17.1%)
Parental smoking	52 (74.3%)
Pets at home	25 (35.7%)
Urban living environment	60 (85.7%)
Positive family history of allergy	34 (48.6%)
Type of FA	IgE-mediated: 69 (98.6%); non-IgE-mediated: 1 (1.4%)
Median number of food allergies/child	2 (1–3)
Main food allergens	Cow’s milk: 21 (30.0%) Egg: 16 (22.9%) Peanut: 14 (20.0%) Peach: 13 (18.6%) Legumes: 13 (18.6%) Tree nuts: 9 (12.9%) Lipid transfer protein sensitization: 6 (8.6%) Fish/shellfish: 4 (5.7%)
FA-related symptoms	Cutaneous: 35 (50.0%) Gastrointestinal: 18 (25.7%) Anaphylaxis: 10 (14.3%) Respiratory: 7 (10.0%)

Data are presented as mean ± standard deviation for normally distributed continuous variables, median (25th–75th percentile) for non-normally distributed continuous variables, and *n* (%) for categorical variables. Age at recruitment is additionally reported with its observed range. Allergen categories were not mutually exclusive. BMI: body mass index; FA: food allergy.

**Table 2:** EDY-Q scores and screening results, food neophobia, and Mediterranean diet adherence in the study population (*n* = 70).

Variable	Possible Score Range	Descriptive Statistic
**EDY-Q total score**	0–6	1.05 (0.40–2.40)
**EDY-Q domains**	—	
Selective Eating	0–6	1.50 (0.67–4.00)
Food Avoidance Emotional Disorder (FAED)	0–6	1.00 (0.00–2.00)
Functional Dysphagia	0–6	0.00 (0.00–2.00)
Weight Problems	0–6	0.00 (0.00–1.00)
Cognitive Distortions related to weight/body shape	0–6	0.00 (0.00–0.00)
**EDY-Q screening for ARFID-related symptoms**	—	
Positive EDY-Q screening	—	3 (4.3%)
Negative EDY-Q screening	—	67 (95.7%)
**Pica-related behaviors (EDY-Q item 13)**	0–6	
No endorsement (score 0)	—	67 (95.7%)
Lower-frequency endorsement (scores 1–3)	—	3 (4.3%)
Recurrent pica-related behavior (score ≥4)	—	0 (0.0%)
**Rumination-related behaviors (EDY-Q item 14)**	0–6	
No endorsement (score 0)	—	49 (70.0%)
Lower-frequency endorsement (scores 1–3)	—	15 (21.4%)
Recurrent rumination-related behavior (scores ≥4)	—	6 (8.6%)
**Food neophobia (ICFNS)**	—	
ICFNS total score	8–40	22.76 ± 2.30
Low food neophobia	—	1 (1.4%)
Moderate food neophobia	—	45 (64.3%)
High food neophobia	—	24 (34.3%)
**Mediterranean diet adherence (KIDMED)**	—	
KIDMED total score	−4 to +12	6.00 (5.00–8.00)
Low adherence	—	7 (10.0%)
Intermediate adherence	—	41 (58.6%)
High adherence	—	22 (31.4%)

Data are presented as median (25th–75th percentile) for non-normally distributed continuous variables, mean ± standard deviation for normally distributed continuous variables, and *n* (%) for categorical variables. Possible score ranges refer to the theoretical ranges of the corresponding instruments. EDY-Q total and domain scores and the individual pica and rumination items range from 0 to 6, with higher scores indicating greater symptom frequency or severity. A positive EDY-Q screening result indicates fulfillment of the questionnaire-based screening criteria for ARFID-related symptoms and does not represent a formal clinical diagnosis. Pica- and rumination-related behaviors were assessed using EDY-Q items 13 and 14, respectively. For both items, any endorsement was defined as a score ≥1, whereas recurrent behavior was operationally defined as a score ≥4, consistent with previous EDY-Q-based studies [28,29]. Scores of 1–3 were classified as lower-frequency endorsement. These categories reflect responses to individual questionnaire items and do not indicate a clinical diagnosis of pica or rumination disorder. Higher ICFNS scores indicate greater food neophobia, whereas higher KIDMED scores indicate better adherence to the Mediterranean diet. ICFNS categories were defined as low (≤17), moderate (18–23), and high (≥24) food neophobia. KIDMED categories were defined as low (≤3), intermediate (4–7), and high (≥8) adherence. ARFID: Avoidant/Restrictive Food Intake Disorder; EDY-Q: Eating Disorders in Youth-Questionnaire; FAED: Food Avoidance Emotional Disorder; ICFNS: Italian Child Food Neophobia Scale; KIDMED: Mediterranean Diet Quality Index for Children and Adolescents.

**Table 3:** Main Spearman correlations between EDY-Q, ICFNS, and KIDMED scores.

Variables	Spearman’s ρ	*p* Value
EDY-Q total score vs. Selective Eating	0.912	<0.001
EDY-Q total score vs. FAED	0.826	<0.001
EDY-Q total score vs. Functional Dysphagia	0.754	<0.001
EDY-Q total score vs. Weight Problems	0.488	<0.001
EDY-Q total score vs. Cognitive Distortions	0.071	0.558
Selective Eating vs. FAED	0.678	<0.001
Selective Eating vs. Functional Dysphagia	0.667	<0.001
Selective Eating vs. Weight Problems	0.415	<0.001
Functional Dysphagia vs. FAED	0.505	<0.001
Functional Dysphagia vs. Weight Problems	0.309	0.009
ICFNS vs. EDY-Q total score	0.040	0.745
ICFNS vs. Cognitive Distortions	0.258	0.031
KIDMED vs. EDY-Q total score	−0.302	0.011
KIDMED vs. Selective Eating	−0.356	0.002
KIDMED vs. FAED	−0.231	0.054
KIDMED vs. Functional Dysphagia	−0.238	0.048
KIDMED vs. ICFNS	0.160	0.185

Spearman’s rank-order correlation coefficients (ρ) are reported. All tests were two-tailed. Correlation analyses were exploratory and interpreted as hypothesis-generating; no adjustment for multiple comparisons was applied. FAED: Food Avoidance Emotional Disorder; EDY-Q: Eating Disorders in Youth-Questionnaire; ICFNS: Italian Child Food Neophobia Scale; KIDMED: Mediterranean Diet Quality Index for Children and Adolescents.

**Table 4:** Qualitative themes, subthemes, descriptive thematic summary and representative quotations.

Theme	Subthemes	Descriptive Thematic Summary	Representative Quotations
1. Anticipatory fear of food and tasting	Perceived food threat; somatic anticipation; uncertainty about hidden ingredients	Food, particularly when unfamiliar or uncertain, was perceived as a potential threat. Fear was often described through bodily sensations and expectations of harm.	“I am always afraid of what I eat because I do not know what is in it” (ID62, female, IgE-mediated allergy to fish and shellfish). “I am afraid I will have an allergic reaction” (ID4, male, IgE-mediated allergy to peach, tree nuts, and strawberry; history of anaphylaxis). “I feel short of breath in my throat. I do not want to eat anything” (ID50, male, IgE-mediated allergy to strawberry and peach).
2. Emotional memory of allergic reactions and generalization of risk	Emotional memory of previous reactions; sensory generalization; symbolic warning signals	Previous allergic reactions were described alongside emotional recall and perceived risk associated with foods of similar appearance, color, texture, or symbolic meaning.	“I had a bad reaction to tuna, and I was afraid” (ID17, female, IgE-mediated allergy to fish). “Anything yellow scares me because maybe it is banana” (ID3, male, IgE-mediated allergy to banana). “Apricot has skin like peach” (ID43, male, IgE-mediated allergy to peach; history of anaphylaxis).
3. Selective avoidance and food rigidity	Refusal of unfamiliar foods; ingredient-related uncertainty; sensory-based rejection	Children often described a restricted food repertoire and a stable tendency to refuse new, unknown, or visually unpleasant foods.	“I do not want to eat rice with spinach, pasta with pumpkin, or zucchini because I do not like the texture” (ID45, female, IgE-mediated allergy to tree nuts, soy, legumes, and lipid transfer proteins). “I do not want to eat almost anything because I do not like things I do not know” (ID66, male, IgE-mediated allergy to soy, peas, lupins, and hazelnuts). “I never eat it because I do not know what they put inside” (ID70, female, IgE-mediated allergy to peanut).
4. Constant reassurance-seeking and decision-making dependence	Caregiver-mediated safety checking; repeated confirmation-seeking; limited food autonomy	Children frequently described asking caregivers to confirm food safety and relying on them when deciding whether to eat.	“I always ask my mother if I can eat it” (ID29, male, IgE-mediated allergy to grape; history of anaphylaxis). “I ask my mother or father whether I can eat it” (ID49, female, IgE-mediated allergy to peanut and peas). “I always ask if there is garlic” (ID58, male, IgE-mediated allergy to garlic; history of anaphylaxis).
5. Ambivalent parental dynamics	Protective reassurance; pressure to taste; inconsistent parental responses	Children described parental responses ranging from reassurance and protection to pressure to taste and acceptance of refusal.	“My mother insists, my father does not” (ID1, male, IgE-mediated allergy to milk, egg, and apple). “Dad says that if I do not want it, it is okay, but mom always says I have to try” (ID69, female, IgE-mediated allergy to legumes; history of anaphylaxis). “If I keep eating nothing, I will stay small forever” (ID50, male, IgE-mediated allergy to strawberry and peach).

Quotations were translated from Italian into English for manuscript purposes. Participant identifiers were anonymized. IgE: immunoglobulin E.

**Table 5:** Joint Display integrating quantitative findings, qualitative themes, representative quotations, and mixed-methods interpretations.

Quantitative Finding	Corresponding Qualitative Theme	Representative Quotation	Integrated Interpretation
Positive EDY-Q screening result for ARFID-related symptoms: 3/70 (4.3%)	Anticipatory fear; emotional memory; selective avoidance	“*I am afraid I will have an allergic reaction*” (ID4)	Qualitative data added contextual depth beyond the categorical EDY-Q screening result and were not interpreted as evidence of additional ARFID cases.
Selective Eating was the highest-scoring EDY-Q domain: median 1.50 (IQR 0.67–4.00)	Selective avoidance and food rigidity	“*I never eat what I do not know*” (ID1)	Quantitative and qualitative findings converged at the construct level, indicating that food selectivity was prominent within the assessed sample.
Functional Dysphagia: median 0.00 (IQR 0.00–2.00)	Somatic anticipation and fear of adverse physical consequences	“*I feel short of breath in my throat. I do not want to eat anything*” (ID50)	Bodily fear described in interviews was conceptually consistent with the EDY-Q Functional Dysphagia domain but did not establish a clinical diagnosis.
ICFNS: moderate food neophobia 45/70 (64.3%); high food neophobia 24/70 (34.3%); no significant correlation with EDY-Q total score	Refusal of unfamiliar foods; sensory generalization of perceived risk	“*Anything yellow scares me because maybe it is banana*” (ID3)	The findings suggested partial overlap but also distinction between food neophobia and EDY-Q-assessed avoidant/restrictive features.
KIDMED was inversely correlated with EDY-Q total score (Spearman’s ρ = −0.302; *p* = 0.011), Selective Eating (ρ = −0.356; *p* = 0.002), and Functional Dysphagia (ρ = −0.238; *p* = 0.048)	Restricted food repertoire; ingredient-related uncertainty	“*I never eat it because I do not know what they put inside*” (ID70)	Qualitative accounts contextualized the observed associations; neither component established that selective eating caused lower diet quality.
No corresponding quantitative measure	Caregiver-dependent reassurance and limited food autonomy	“*I always ask my mother if I can eat it*” (ID29)	The qualitative component identified a relational dimension not captured by the questionnaires, representing complementarity between methods.

The qualitative subsample was purposively selected; therefore, quotations and themes were used for interpretative integration and not for estimating frequencies in the full sample. EDY-Q: Eating Disorders in Youth-Questionnaire; FAED: Food Avoidance Emotional Disorder; ICFNS: Italian Child Food Neophobia Scale; KIDMED: Mediterranean Diet Quality Index for Children and Adolescents.

## Data Availability

The data presented in this study are available on reasonable request from the corresponding author, in accordance with applicable ethical and privacy regulations.
